# Differences in Male-Killing *Rickettsia* Bacteria between Lineages of the Invasive Gall-Causing Pest *Leptocybe invasa*

**DOI:** 10.3390/insects14090757

**Published:** 2023-09-11

**Authors:** Xin Peng, Hantang Wang, Zhende Yang

**Affiliations:** Guangxi Colleges and Universities Key Laboratory for Cultivation and Utilization of Subtropical Forest Plantation, Guangxi Key Laboratory of Forest Ecology and Conservation, College of Forestry, Guangxi University, Nanning 530004, China; 1909302008@st.gxu.edu.cn (X.P.); 1909302013@st.gxu.edu.cn (H.W.)

**Keywords:** *Leptocybe invasa*, *Rickettsia*, lineage identification, 16S rRNA gene

## Abstract

**Simple Summary:**

This study compared the differences in symbiotic bacteria between the A and B lineages of the *Leptocybe invasa*, so as to provide a reference for the scientific control of the pest.

**Abstract:**

(1) Background: *Leptocybe invasa* (Hymenoptera: Eulophidae) is a global invasive pest that seriously damages eucalyptus plants and has caused serious harm to forestry production in many countries. Two genotypically distinct lineages of *L. invasa* have been detected outside of Australia, namely, lineage A and lineage B. However, the composition and abundance of endosymbiotic bacteria in *L. invasa* are still unclear between lineages. Therefore, the purpose of this study was to compare the bacterial communities in female adults of *L. invasa* of different lineages distributed in the same domain; (2) Methods: The PacBio Sequel II platform was used to compare bacterial community composition between lineages of *L. invasa* by sequencing the V1–V9 region of the 16S rRNA gene, and fluorescence quantitative PCR was used to compare the relative expression of *Rickettsia* between lineages of *L. invasa*; (3) Results: A total of 437 operational taxonomic units (OTUs) were obtained. These OTUs were subdivided into 20 phyla, 32 classes, 77 orders, 129 families, and 217 genera. At the genus level, the dominant bacteria in lineage A and lineage B were *Rickettsia* and *Bacteroides*, respectively. There were differences in the bacterial community of *L. invasa* between lineages, and the abundance and relative expression of *Rickettsia* in lineage A were significantly higher than those in lineage B; (4) Conclusions: There were differences in the bacterial community of *L. invasa* between lineages, and the abundance and relative expression of *Rickettsia* in lineage A were significantly higher than those in lineage B.

## 1. Introduction

Insects generally carry heritable endosymbiotic bacteria [1], and the main mode of transmission is maternal inheritance [2]. The interaction between endosymbiotic bacteria and insects is often complex. In the long-term process of coevolution, endosymbiotic bacteria generally live in host-specific bacteria-containing cells, and the host insects provide them with the necessary ecological conditions for growth. Endosymbiotic bacteria can impart new biological characteristics on host insects [3] and play important roles in host growth and development, reproductive regulation, environmental adaptation, genetic evolution, immune responses, and many other biological processes [4,5,6,7]. They are interdependent, interactive, and coevolutionary [8]. For example, *Rickettsia* can shorten the generation development period and improve the survival, egg laying, and offspring sex ratio in whitefly [9,10]. *Wolbachia*, an endosymbiotic bacterium, can enhance mosquitoes’ resistance to RNA viruses [11,12]. In recent years, pest control by eliminating endosymbiotic bacteria has been successfully applied. For example, the mixed use of antibiotics and insecticides can effectively reduce the number of endosymbiotic bacteria in *Nilaparvata lugens Stal* and improve the control effect of insecticides [13]. By eliminating the endosymbiotic bacterium *Wolbachia* from mosquitoes, mosquito fecundity is effectively reduced [14]. Therefore, understanding of the composition and functions of bacteria in insects is of great significance for pest control.

*Leptocybe invasa* Fisher & LaSalle (Hymenoptera: Eulophidae) is a globally invasive pest that seriously damages *eucalyptus* plants. *L. invasa* female adults lay their eggs in eucalyptus branches and leaves to reproduce their offspring, resulting in lumpy galls on the leaf veins, petioles, and branches that have serious effects on the normal growth of the plant [15]. The main reproductive mode of *L. invasa* is parthenogenesis [16], which makes the success rate of invasion and colonization very high. In addition, the amazing reproductive rate and population numbers also enable *L. invasa* to break out rapidly in areas invaded, causing large economic losses to forestry production in many countries. *L. invasa* was first discovered in the Middle East and Mediterranean in 2000 [16]. It had spread to 29 countries on five continents by 2014, and that number soared to 45 countries in 2018 [16,17,18]. In China, *L. invasa* was first found in Dongxing city, Guangxi Zhuang Autonomous Region, on the border with Vietnam, in 2007 [19] and has now spread to Guangxi, Hainan, Guangdong, Fujian, Sichuan, Jiangxi, Yunnan, and Taiwan Provinces [18,20]. Under the scenario of future climate warming, the potential core distribution area of *L. invasa* in China will be located in Yunnan, Guangxi, Guangdong, and Hainan Provinces and will spread to high latitudes (Hubei, Anhui, Zhejiang, Jiangsu, etc.) [21].

Human activities have broken the original barriers of geographical isolation of pests and led to the mixing of pests of different lineages, which has created new challenges for pest management. The invasion of pests in the form of lineage complexes may be the fundamental reason for the repeated occurrence of pests and the failure in some areas to control pests [22], such as *Gonipterus scutellatus* [22], *Bemisia tabaci* [23], and *Cacopsylla chinensis* [24]. Mitochondrial COI sequence data have shown that *L. invasa* has three lineages, A, B (B1 and B2), and C, of which lineages B2 and C only appear in Australia, while lineages A and B1 are widely distributed in areas invaded by *L. invasa*. *L. invasa* lineages A and B1 coexist in China, Laos, Vietnam, Thailand, and South Africa (lineage B in this paper refers to lineage B1) [15,25,26,27].

To date, there have been many reports on the bacteria in *L. invasa*. Nugnes et al. [25] found that *Rickettsia* in *L. invasa* is located in the ovary of female adults, indicating that *Rickettsia* is related to its parthenogenesis. Guo et al. [28] found that the content of *Rickettsia* in male *L. invasa* was much lower than that in female *L. invasa*, and the difference in *Rickettsia* content between males and females may be due to the difference in their physiological structure. Liu et al. [29] identified the types of culturable bacteria in *L. invasa* at different developmental stages and identified 88 kinds of bacteria belonging to 4 phyla, 27 families, and 44 genera, of which 72 kinds were isolated at the gall stage and 46 kinds at the adult stage. The most abundant bacterial species was γ-Proteobacteria. There were significant differences in total number of bacteria and community composition among developmental stages of *L. invasa*. Guo et al. [30] studied the bacterial diversity in 10 geographical populations of *L. invasa* in China and found that differences in bacterial species and abundances among geographical populations, with *Rickettsia* dominant in most populations. Although these studies have clarified the bacterial diversity and function of *L. invasa* from many angles, deficiencies remain because most researchers ignored the influence of lineage type on the symbiotic bacteria of *L. invasa* before carrying out the research. Even as early as 2015, Nugnes et al. proved that *Rickettsia* in lineages A and B of *L. invasa* had differentiated genetically and speculated that there may be differences in reproductive strategy between lineages A and B. Therefore, clarifying the bacterial composition and abundances in different lineages of *L. invasa* can supplement and verify many of the research results described above.

## 2. Materials and Methods

### 2.1. Sample Collection

The samples used in this study were collected from Luchuan City, Guangxi Zhuang Autonomous Region, China (110°15′ E, 22°17′ N) in November 2021. The host plant was DH201-2: Eucalyptus grandis × Eucalyptus tereticornis (Myrtales: Myrtaceae). Fresh eucalyptus shoots with galls were collected from woodland, and the branches were placed in a plastic bottle filled with water to maintain freshness and then transferred into a sealed net cage (40 cm × 40 cm × 80 cm) at room temperature to keep the adults from escaping. The water in the plastic bottle was renewed daily until *L. invasa* adults emerged. Sexes were identified by morphological observation. Female *L. invasa* adults were selected as test insects within 12 h of emergence.

### 2.2. Sample Processing and Extraction of L. invasa Total Genomic DNA

A total of 200 adult female *L. invasa* were collected within 12 h of emergence and starved for 12 h. The surface of each *L. invasa* female was disinfected with 75% alcohol 3 times in an ultraclean workbench for 30 s each time and then rinsed with sterile water 5 times. Finally, the head of each *L. invasa* female was cut off in sterile water. The separated head, chest, and abdomen of each female were placed in a sterilized 1.5 mL centrifuge tube, marked, and stored in a refrigerator at −20 °C until use. An animal tissue DNA extraction kit (column type, Beijing Qingke Biotechnology Co., Ltd. Beijing, China) was used to extract the total genomic DNA of female *L. invasa* heads. According to the manufacturer’s instructions, the quality and concentration of DNA were determined at ratios of 260 nm/280 nm and 260 nm/230 nm using a Nanodrop 2000 Ultra-micro UV spectrophotometer. Qualified DNA was stored in a −20 °C refrigerator until use.

### 2.3. Identification of L. invasa Lineages

Extracted *L. invasa* DNA templates were amplified by PCR with mtDNA COI universal primers LCO1490 (5′-GGTCAACAAATCATAAAGATATTGG-3′) and HCO2198 (5′-TAAACTT CAGGGTGACCAAAAAATCA-3′) [31]. Each PCR system contained 25.0 μL, including 12.5 μL of 2 × Hotstart PCR Master Mix (without dye) (Shanghai Shenggong Bioengineering Technology Service Co., Ltd. Shanghai, China), 0.5 μL (10 μM) of each of the forward and reverse primers, 2.0 μL of DNA template, and 9.5 μL of ddH_2_O. The PCR procedure was predenaturation at 95 °C for 3 min; 30 cycles of denaturation at 95 °C for 20 s, annealing at 49 °C for 20 s, and extension at 72 °C for 10 s; and a final extension at 72 °C for 3 min. PCR products were mixed with 1 μL of 6× loading buffer and detected by 1% agarose gel electrophoresis. Each gel was then cut, purified, and sent to Shanghai Shenggong Bioengineering Technology Service Co., Ltd., Shanghai, China for sequencing. The COI sequence for the *L. invasa* were downloaded from the GenBank molecular database (ON166667-ON166668, MH093001-MH093481, JQ289999-JQ290005, KP233972-KP233993, KP233954, and MZ378835-MZ379154). The COI sequence GenBank accession numbers obtained in this study were ON166667 (Lineage A) and ON166668 (Lineage B) (because the COI gene sequence is consistent in the *L. invasa* lineage, we only uploaded one sequence of each lineage). Using MEGAX v7.0 software, all COI sequences were compared, and a sample neighbor-joining (NJ) phylogenetic tree was established with the Kimura 2-parameter model. *L. invasa* lineages were identified according to the results of the phylogenetic tree. When the number of female *L. invasa* adults of lineages A and B to be identified reached 50, their chests and abdomens were mixed according to lineage type in an ultraclean workbench.

### 2.4. Extraction of Total Microbial DNA from L. invasa and Amplification of the 16S rRNA Gene Sequence

*L. invasa* lineage A and B samples were ground with liquid nitrogen, and the powder was placed in sterile centrifuge tubes. The PowerSoil^®^ DNA Isolation kit was used to extract the total microbial DNA of each lineage sample. The quality and concentration of DNA were detected by a Nanodrop 2000 Ultra-micro UV spectrophotometer. Primers 27F: 5’-AGRGTTTGATYNTGGCTCAG-3’ and 1492R: 5’-TASGGHTACCTTGTTASGACTT-3’ were used to amplify the full length sequence of 16S rRNA gene (V1-V9) [32]. The sequencing library was obtained according to the preparation process for the 16S Amplification SMRTbell^®^ library, and the library was sequenced on the PacBio Sequel II platform [33]. Database construction and high-throughput sequencing were completed by Beijing Biomarker Technologies Co., Ltd., Beijing, China (GenBank registered number: PRJNA845099).

### 2.5. Data Optimization and Statistical Analysis

According to the barcode sequence of each sample, Limma 1.9.0 software was used to separate sample data from the off-board data. After correction by CCS v3.4.1 software, the sequences that were too long or too short and those with too few subreads were filtered out to obtain raw reads. Finally, primer excision, simple sequence repeat (SSR) removal, and chimera removal were carried out to obtain clean FASTQ files. UPARSE (usearch 10) software was used to cluster the filtered data for all samples into operational taxonomic units (OTUs) according to a 97% similarity level. Diversity analysis was then carried out, and the alpha diversity index was calculated by QIIME 1.9.1 software to evaluate the species richness and diversity in each single sample. Differences in community structure and species abundances between samples were analyzed by counting the species with relative abundance greater than 1%.

### 2.6. Phylogenetic Analysis of Rickettsia Endosymbionts from L. invasa Based on 16S rRNA Gene Sequences

The GenBank accession numbers of *Rickettsia* 16S rRNA full length gene sequences from *L. invasa* lineages A and B obtained in this study were OR466117 (lineage A) and OR466118 (lineage B), respectively (1421 bp, because the *Rickettsia* 16S rRNA gene sequence is consistent within *L. invasa* lineages, we uploaded only one sequence from each lineage). Then, we downloaded 16S rRNA v3–v4 region gene sequences of *L. invasa* from various regions around the world, as well as 16S rRNA gene sequences of different lineages of *Rickettsia*, from the GenBank database. We used MEGAXv7.0 software to align and trim all 16S rRNA gene sequences, resulting in 21 sequences of 744 bp length for the 16S rRNA gene. Subsequently, we employed MEGAXv7.0 software to construct a neighbor-joining (NJ) phylogenetic tree using the Kimura 2-parameter model.

### 2.7. Fluorescence Quantitative PCR Verification

This experiment utilizes *L. invasa* β-Actin as the reference gene and the gltA gene of the symbiotic bacterium *Rickettsia* as the target gene. Primers and probes have been designed accordingly, and the SYBR GreenI method is employed to assess the infection density of *Rickettsia* within *L. invasa*. The primers used for amplifying the gltA gene of *Rickettsia* in this experiment are gltA-F: 5′-AG AATTACCAAGCATCGAGCAG-3′and gltA-R: 5′-GCCGCAAGCATAATAGCCATA-3′. The primers for the β-Actin gene are β-Actin-F: 5′-GCCGTGTTCCCGTCCATCGT-3′ and β-Actin-R: 5′-GCGGTTGCCATTT CCTGCTC-3′ [34].

Using the ChamQ Universal SYBR qPCR Master Mix fluorescence quantification kit, the quantitative analysis of *Rickettsia* was performed on 20 samples from each lineage (A and B) of *L. invasa*, following the instructions provided in the user manual. Quantitative real-time reverse transcription polymerase chain reaction (qRT-PCR) reactions were conducted using the LightCycler^®^ 480 real-time fluorescence quantification PCR system. The reaction mixture consisted of 10.0 µL of 2×ChamQ Universal SYBR qPCR Master Mix, 0.4 µL of forward primer, 0.4 µL of reverse primer, and 1 µL of template DNA, and was adjusted to a total volume of 20 µL with ddH_2_O. The qRT-PCR reaction program comprised an initial denaturation step at 95 °C for 30 s, followed by 40 cycles of denaturation at 95 °C for 10 s, annealing at 60 °C for 30 s, and extension. The melt curve analysis was performed with the following conditions: denaturation at 95 °C for 15 s, annealing at 60 °C for 60 s, and denaturation at 95 °C for 30 s. Following the completion of the reaction, the primer specificity of the qRT-PCR was assessed through melt curve analysis. The reliability of the chosen reference gene and primer design was determined based on the standard curve. The cycle threshold (Ct) values for the gltA gene of *Rickettsia* and the β-Actin gene of *L. invasa* were recorded. The expression level of the symbiotic bacteria *Rickettsia* within *L. invasa* was calculated using the 2-ΔΔCt method.

## 3. Results

### 3.1. Identification of L. invasa Lineages

**Figure 1:**
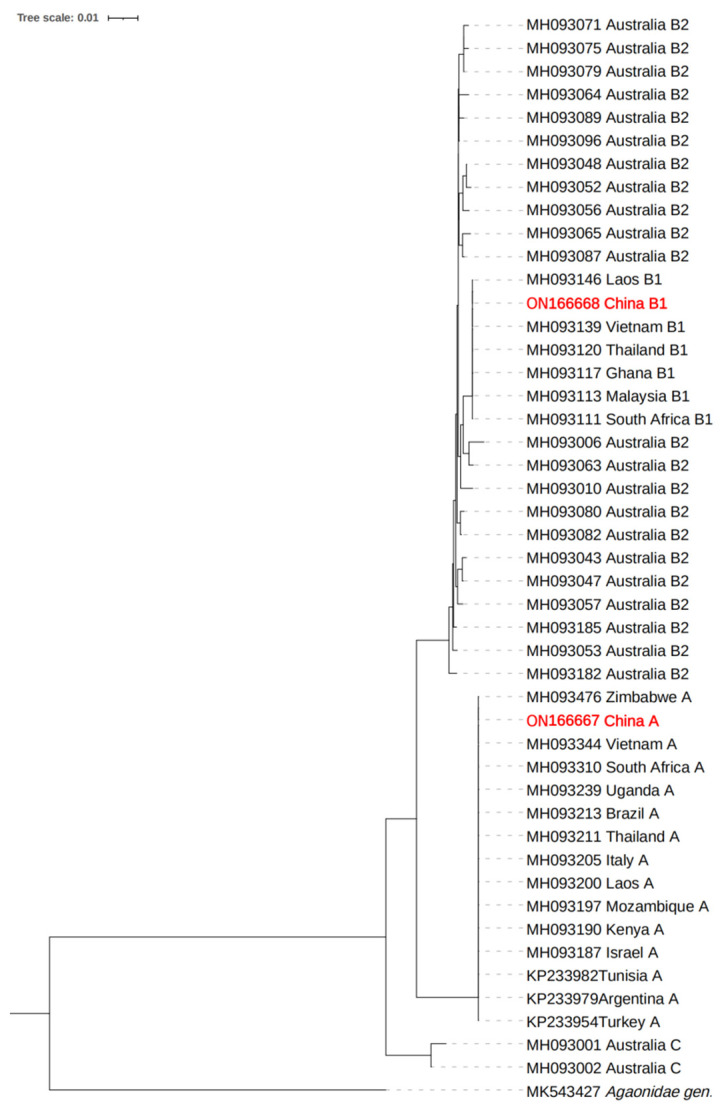
The NJ phylogenetic tree of COI sequence of *L. invasa*.

The NJ phylogenetic tree based on mitochondrial COI sequence data showed that the adult female *L. invasa* sampled in this study belonged to lineage A and lineage B, and the mitochondrial COI sequence is consistent within each *L. invasa* lineage (Figure 1).

### 3.2. Bacterial Diversity and Abundance in L. invasa Lineages A and B

**Figure 2:**
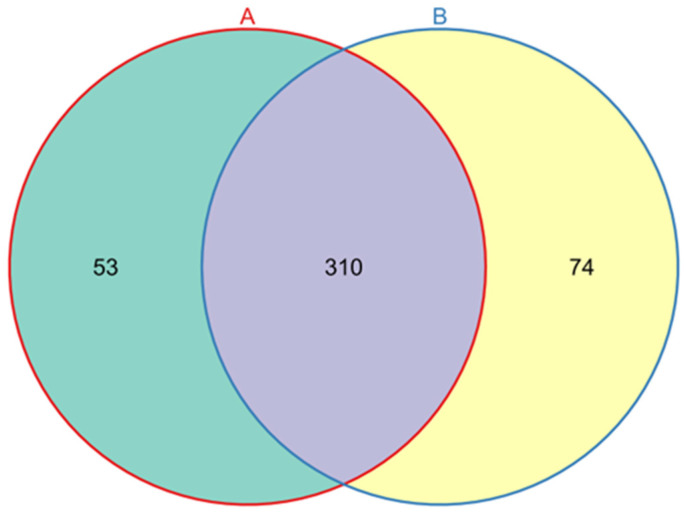
Venn diagram of OTUs of endosymbiotic bacteria found in lineage A and lineage B of *L. invasa*.

A total of 15,038 high-quality 16S rRNA gene sequences were generated from *L. invasa* lineage A and B samples, and an average of 7519 high-quality 16S rRNA gene sequences were generated from each sample. After grouping OTUs according to sequence similarity, a total of 437 OTUs were obtained. A total of 363 and 384 OTUs were obtained for the lineage A and B samples, respectively, including 53 OTUs unique to lineage A and 74 OTUs unique to lineage B (Figure 2). There were 20 phyla, 32 classes, 77 orders, 129 families, 217 genera, and 261 species noted (Table 1).

**Figure 3:**
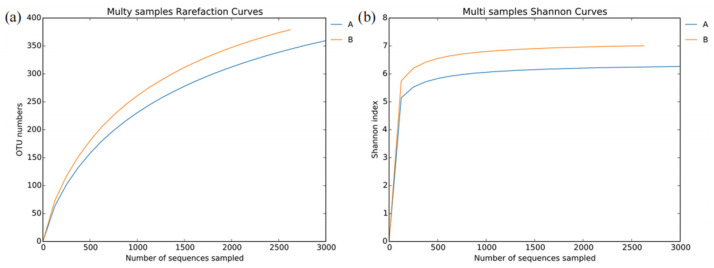
Dilution curves (**a**) and Shannon index curves (**b**) of the number of bacterial OTUs observed in *L. invasa*.

The dilution curve and Shannon index curve were used to evaluate whether the numbers of samples sequenced were sufficient. The results are shown in Figure 3. The dilution curves indicate that the samples of *L. invasa* lineages A and B have not entered the plateau stage, which suggests it is possible to find new bacterial species with increasing sequencing effort, but the Shannon index curves indicate that the samples of *L. invasa* lineages A and B have entered the plateau stage, suggesting that the diversity of bacteria in the samples can be fully displayed at this sequencing depth. Therefore, the degree of bacterial sequencing in this study meets the requirements of subsequent bioinformatics analysis.

Alpha diversity was estimated by five indices: Chao1, Shannon index, Simpson index, ACE, and coverage (Table 2). The Chao1 (447.7917 vs. 462.2692) and ACE (460.7090 vs. 474.4288) values of lineage A were lower than those of lineage B. There was also good consistency between the Simpson index and Shannon index, indicating that the diversity of the bacterial community of lineage B was higher than that of lineage A. The coverage of *L. invasa* lineages A and B was higher than 95%, indicating that the probability of detecting bacteria was much higher than that of not detecting bacteria.

### 3.3. Bacterial Community Structure in L. invasa Lineages A and B

**Figure 4:**
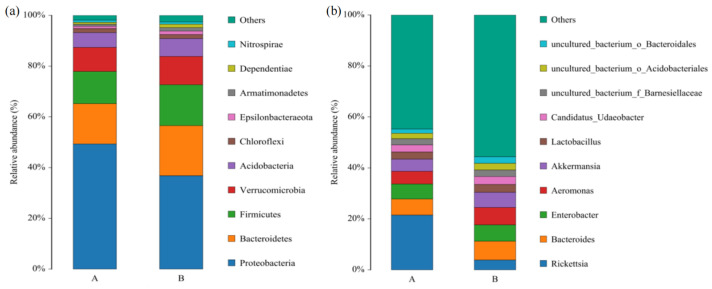
Relative abundances of the top 10 bacterial phyla (**a**) and genera (**b**) in lineage A and lineage B of *L. invasa*.

At the phylum level, the bacteria in *L. invasa* lineages A and B mainly belonged to Proteobacteria, Bacteroidetes, and Firmicutes, and their relative abundances were 49.34%, 36.81%, and 15.87%, respectively. In addition, bacteria with abundance greater than 1% also included Verrucomicrobia, Acidobacteria, and Chloroflexi (Figure 4a).

At the genus level, the dominant bacteria in *L. invasa* lineage A mainly included *Rickettsia*, *Bacteroides*, *Enterobacter*, *Aeromonas*, and *Akkermansia*, with abundances of 21.50%, 6.27%, 5.92%, 4.98%, and 4.78%, respectively. The dominant bacteria in lineage B mainly included *Bacteroides*, *Aeromonas*, *Enterobacter*, *Akkermansia*, and *Rickettsia*, with abundances of 7.37%, 6.82%, 6.42%, 5.95%, and 3.87%, respectively. The bacterial species in *L. invasa* lineages A and B were essentially identical, but there was a significant difference in the relative abundance of *Rickettsia* (Figure 4b).

### 3.4. Phylogenetic Analysis of L. invasa Rickettsia Based on 16S rRNA Gene Sequence

**Figure 5:**
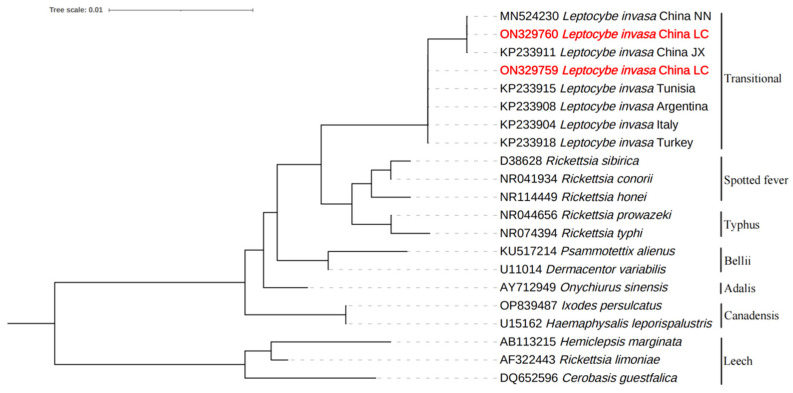
Phylogenetic analysis of *L. invasa Rickettsia* based on 16S rRNA gene sequences.

The phylogenetic tree based on *Rickettsia* 16S rRNA gene sequences showed *Rickettsia* inhabiting *L. invasa* lineage A and lineage B to be differentiated (Figure 5). By searching and comparing the original data for all 16S rRNA gene sequences of each lineage, no mixing of *Rickettsia* from *L. invasa* lineages A and B was found.

### 3.5. Fluorescent Quantitative PCR Validation

**Figure 6:**
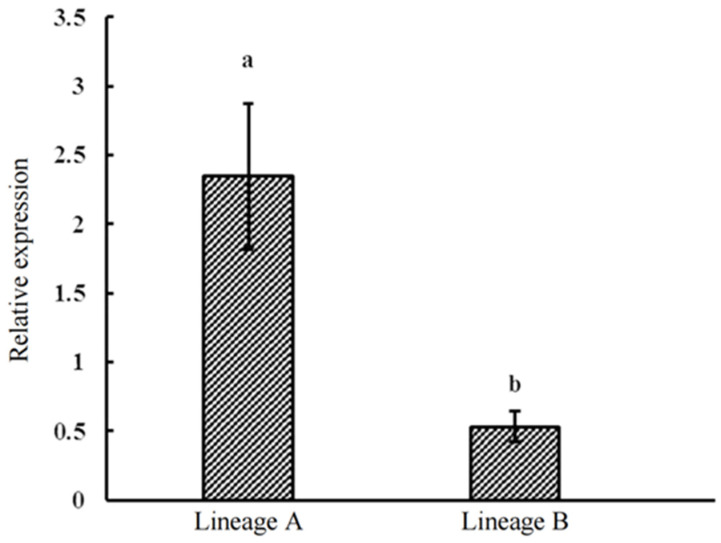
Relative expression of *Rickettsia* from different lineages of *L. invasa*.

The obtained melting curves for the gltA gene of *Rickettsia* and the β-Actin gene of *L. invasa* through qRT-PCR exhibit singular peaks, characterized by narrow and sharp peak lines. This indicates a high level of primer specificity for the two amplified genes in qRT-PCR, resulting in a single amplification product. Ct values were obtained after qRT PCR amplification of continuously diluted DNA of different concentrations and draw standard curves (Supporting Information). Through the investigation of the β-Actin gene of *L. invasa* lineages A (20 individuals) and B (20 individuals), as well as the *Rickettsia* gltA gene, the results reveal the presence of *Rickettsia* within the bodies of both A and B lineages of *L. invasa* female adults. Based on the results of a univariate analysis, it was found that the relative expression level of *Rickettsia* within the bodies of the *L. invasa* varies between different lineages. Specifically, the relative expression level (a,b) of *Rickettsia* in lineage A (2.35 ± 0.53) is significantly higher than in lineage B (0.53 ± 0.11) (Figure 6), which is consistent with the high-throughput sequencing results.

## 4. Discussion

The genetic characteristics of insects affect the species of endosymbiotic bacteria they host [35]. For example, *Acyrthosiphon pisum* populations of the same biotype carry similar endosymbiotic bacterial communities, while the composition of endosymbiotic bacterial communities varies among biotypes [36]. *Bemisia tabaci* lineage B in Israel mainly hosts *Rickettsia* and *Hamiltonella*, while *Bemisia tabaci* lineage Q mainly hosts *Rickettsia* and *Arsenophonus*, and a few individuals are infected with *Wolbachia* [37]. Chu et al. [38] examined 24 *Bemisia tabaci* populations and found that *Wolbachia* was not detected in *Bemisia tabaci* populations of lineages B and Q, but *Wolbachia* was detected in the Zhejiang population in China and Kenya populations other than lineages B and Q, and *Wolbachia* in the *Bemisia tabaci* populations in Zhejiang and Kenya belonged to different genotypes. This study found that both *L. invasa* lineages A and B contained *Rickettsia*, but *Rickettsia* was differentiated between lineages, which was consistent with the results of Nugnes et al. [25].

Endosymbiotic bacteria in insects often spread in a strict vertical matrilineal manner, that is, from female carrier insects to offspring through eggs [39]. Nugnes et al. [25] found that *Rickettsia* in *L. invasa* is located in the ovary of female adults and believed that *Rickettsia* is the main cause of parthenogenesis. Results reported by Guo et al. [28] also supported this conjecture; the abundance of *Rickettsia* in male *L. invasa* was much lower than that in female *L. invasa*, and this difference in *Rickettsia* abundance between males and females may stem from differences in their physiological structure. Shan et al. [40] also revealed the pathway and mechanism by which *Rickettsia* reaches and enters the offspring during the oogenesis and embryonic development of the host insect. It is worth mentioning that not all transmission of symbiotic bacteria among insects is vertical, and a low incidence of horizontal transmission also occurs [41]. The proposed horizontal transmission routes mainly include plant-mediated horizontal transmission, parasitic wasp-mediated horizontal transmission, and sexual mating-mediated horizontal transmission [42]. This study sequenced and analyzed the microorganisms in *L. invasa* lineages A and B distributed in the same domain. It was found that different types of *Rickettsia* did not mix between lineages, indicating that *Rickettsia* in *L. invasa* showed strict maternal inheritance.

The genetic characteristics of host insects affect the abundance of endosymbiotic bacteria. For example, the population abundance of Buchnera in *Acyrthosiphon pisum* is affected by lineage [43]. The abundance of *Wolbachia* in *Leptopilina heterotoma* has also been found to be significantly affected by its genotype, with the role of host insect genotype related to ambient temperature [44]. The abundances of the two endosymbiotic bacteria *Moranella* and *Tremblaya* in *Planococcus citri* are affected by the genotype of the host insect, and the abundances of the endosymbiotic bacterial populations are determined by maternal inheritance of the host pest [45]. This study found that in the Luchuan population in Guangxi, the abundance and relative expression of endosymbiotic bacterium *Rickettsia* in *L. invasa* lineage A were significantly higher than those in lineage B. We believe that these differences derive from the differences in genetic characteristics between lineages.

In addition, different geographical populations or climatic conditions also lead to differences in the species of endosymbiotic bacteria in host insect populations. Guo et al. [30] studied the bacterial diversity of *L. invasa* in 10 geographical populations in China and found differences in the bacterial diversity and abundance of *L. invasa* among geographical populations. *Rickettsia* was dominant in most populations, but the abundances of *Rickettsia* in Deyang, Sichuan, Qinzhou, Guangxi, and Sanming, Fujian, were significantly lower than those of other populations. The *L. invasa* samples used in this study were collected from Luchuan City, Guangxi Province, and the abundance of *Rickettsia* was detected to be low, which may be related to the sampled population in the field.

Dittrich-Schröder et al. [15] studied the population genetics of *L. invasa* around the world and found introgressive hybridization between *L. invasa* lineages A and B in Vietnam. Peng et al. [27] conducted population genetic research on 14 geographical populations of *L. invasa* in China and found that 7 geographical populations included both lineages A and B. Of these, there was obvious introgressive hybridization between lineages in Wuzhou, Guangxi, and Panzhihua, Sichuan. The genetic diversity of *L. invasa* lineage A is significantly higher than that of lineage B. These researchers believe that this is caused by the introgression of a large number of genes into lineage A from *L. invasa* lineage B and speculate that *L. invasa* lineage B may be more inclined to sexual reproduction. With the reports of introgressive hybridization between *L. invasa* lineages, the differences in reproductive strategies between *L. invasa* lineages have attracted much attention. Previous studies have also shown that male *L. invasa* adults have never been reported in areas where *L. invasa* lineage A has invaded, such as Italy, Tunisia, or Argentina, and there are few males in Turkey (male to female ratio 0–0.5%), while male *L. invasa* adults appear more frequently in areas where *L. invasa* lineage B has invaded, such as Thailand, India, and China (7.2%), so it is speculated that lineage B tends to produce males [25,28]. In Hymenoptera, where parthenogenesis is the main mode of reproduction, a threshold density of *Rickettsia* bacteria in eggs is necessary to trigger the development of female embryos, and the phenomenon of male parthenogenesis may occur when *Rickettsia* is removed with antibiotics. For example, *Pnigalio soemius*, which employs parthenogenesis as the main reproductive mode, produces male offspring 24 h after consuming 20 mg/mL rifampicin [46]. Therefore, we believe that differences in *Rickettsia* abundance between lineages of *L. invasa* lead to differences in reproductive strategy between lineages. The low abundance of *Rickettsia* in lineage B may lead to higher numbers of males and a greater tendency towards sexual reproduction, while the high abundance of *Rickettsia* in lineage A may make this lineage more inclined towards parthenogenesis, so it is more dominant in invasive colonization.

There are obvious differences in genetics and endosymbiotic bacteria between lineages of *L. invasa*. We suggest that it is necessary to rethink the research conclusions regarding *L. invasa* and the effectiveness of control measures. It is urgent for researchers to distinguish lineages and clarify the research target species before selecting control measures or conducting physiological and biochemical studies to eliminate the impact of differences in lineages on research results.

## 5. Conclusions

*Rickettsia* exhibited genetic differentiation between *L. invasa* lineages. The abundance and relative expression of *Rickettsia* in lineage A were significantly higher than those in lineage B.

## Figures and Tables

**Table 1:** Statistics of classification levels for endosymbiotic bacteria found in *L. invasa* lineages A and B.

Lineage Type	Phylum	Class	Order	Family	Genus	Species
Lineage A	18	29	71	112	185	226
Lineage B	20	31	76	125	204	240
Lineage A-specific	0	1	1	4	13	21
Lineage B-specific	2	3	6	17	32	35
Lineage in common	18	28	70	108	172	205
Total	20	32	77	129	217	261

**Table 2:** Statistics of alpha diversity indices of the bacteria in female adults of *L. invasa* lineages A and B.

Lineage Type	ACE	Chao 1	Shannon	Simpson	Coverage
Lineage A	460.7090	447.7917	6.269	0.9419	0.9641
Lineage B	474.4288	462.2692	7.013	0.9803	0.9595

## Data Availability

The data that support the findings of this study are openly available in the NCBI data repository by searching for accession numbers ON166667-ON166668, OR466117- OR466118 and PRJNA845099.

## Supplementary Materials

The following supporting information can be downloaded at: https://www.mdpi.com/article/10.3390/insects14090757/s1, Figure S1: The Standard curve of *gltA* gene.
