# Supplementary figures and images for: Differences in Male-Killing Rickettsia Bacteria between Lineages of the Invasive Gall-Causing Pest Leptocybe invasa
Source: Insects. 2023 Sep 11;14(9):757. doi: 10.3390/insects14090757 (PMC10532318; doi:10.3390/insects14090757)

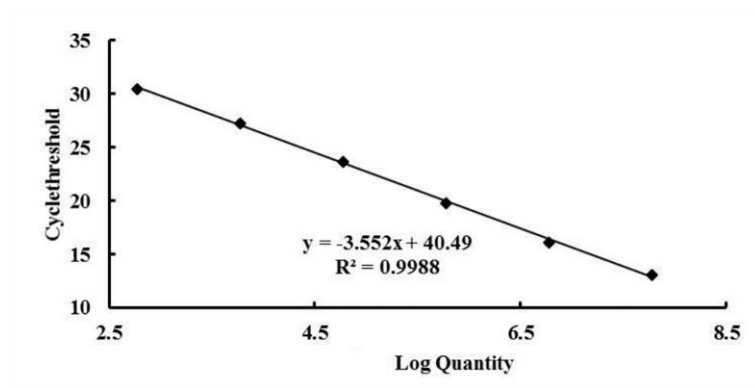

**Figure S1.** The Standard curve of *gltA* gene.

Supplement: Supplementary file 1 [file insects-14-00757-s001.zip › insects-2525161-supplementary.pdf]
